# Boosting AMPs’ Power: From Structural Engineering to Nanotechnology-Based Delivery

**DOI:** 10.3390/molecules30142979

**Published:** 2025-07-15

**Authors:** Oluwasegun Eric Ajayi, Rosa Bellavita, Lorenzo Emiliano Imbò, Sara Palladino, Simone Braccia, Annarita Falanga, Stefania Galdiero

**Affiliations:** 1 INTHERAPI Graduate School, Universite de Bourgogne, 21000 Dijon, France; ae.oluwasegun@gmail.com; 2 Department of Pharmacy, School of Medicine, University of Naples Federico II, Via Domenico Montesano 49, 80131 Napoli, Italy; rosa.bellavita@unina.it (R.B.); sara.palladino@unina.it (S.P.); simone.braccia@unina.it (S.B.); 3 Department of Agricultural Sciences, University of Naples Federico II, Via Università 100, Portici, 80055 Portici, Italy; lorenzoemiliano.imbo@unina.it (L.E.I.); annarita.falanga@unina.it (A.F.)

**Keywords:** antimicrobial peptides, drug delivery, peptide strategies, nanoparticle conjugation, biofilms

## Abstract

Antimicrobial peptides (AMPs) represent a powerful support to conventional antibiotics in addressing the global challenge of antimicrobial resistance (AMR). Their broad-spectrum antimicrobial activity and unique mechanisms of action enable diverse potential applications, including combating multidrug-resistant pathogens, immune modulation, and cancer therapy. Their clinical implementation is hindered by challenges such as toxicity, instability, and high production costs. Recent advances in AMP design, optimization, and delivery mechanisms such as nanoparticle conjugation and rational engineering have enhanced their efficacy, stability, and specificity. Integrating AMPs into precision medicine and combining them with existing therapies promises to overcome current limitations. With ongoing advancements, AMPs have the potential to redefine infection management and possibly other medical problems.

## 1. Introduction

**Figure 1:**
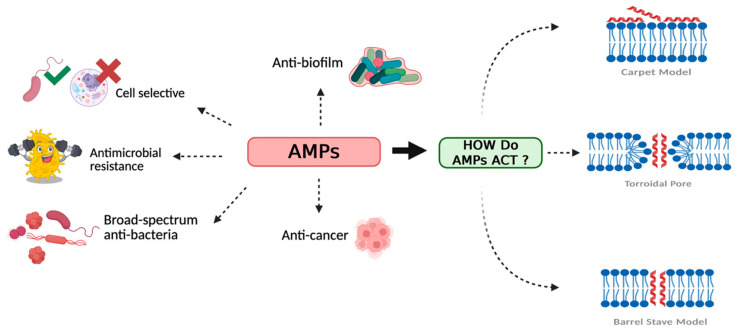
Schematic representation of the main activities and mechanisms of action of AMPs. The figure was created with https://app.biorender.com/ (Accessed on 20 June 2025).

Antimicrobial resistance (AMR) is a critical global health and economic issue, with the World Health Organization (WHO) urging immediate action to combat it [1]. The growing occurrence of drug-resistant bacteria and fungi, including the ESKAPE pathogens (*Enterococcus faecium*, *Staphylococcus aureus*, *Klebsiella Pneumoniae*, *Acinetobacter baumannii*, *Pseudomonas aeruginosa*, and *Enterobacter* spp.), has made AMR a top priority, especially in low- and middle-income countries [2,3,4]. The overuse and misuse of conventional antibiotics accelerate resistance, making them ineffective in treating ESKAPE infections but also leading to significant side effects, including toxicity and microbiome disruption [4]. These challenges highlight the urgent need for alternative strategies, such as the use of antimicrobial peptides (AMPs) [5,6], which present a unique mechanism of action and versatility, making them a promising approach [7]. They are small, cationic peptides playing a key role in the innate immune systems of various organisms, including humans, animals, and plants. Microorganisms produce AMPs as part of their competitive defense, with species-specific peptides offering unique antimicrobial properties [5], which makes them essential natural compounds in the fight against infections. AMPs act as a rapid and non-specific defense mechanism, capable of destroying pathogens by interacting with and damaging their membranes [1,2]. In addition, some AMPs self-assemble into fibrillar, amyloid-like nanostructures, enhancing their antimicrobial potential [3]. The diversity of AMPs reflects their evolutionary role across life forms. Animals, including amphibians, mammals, reptiles, and arthropods, are rich sources of AMPs. Mammals produce AMPs in glandular secretions as part of their immune defense [8]. Amphibians like *Xenopus laevis* produce magainin in skin secretions, while aquatic species such as horseshoe crabs (*Tachypleus tridentatus*) produce tachyplesin, and shrimp (*Litopenaeus vannamei*) synthesize penaeidin [8]. Similarly, plants produce AMPs in leaves, stems, and fruits to combat microbial infections [9]. AMPs exhibit a broad spectrum of activity due to their ability to disrupt microbial lipid bilayers, leading to leakage of cell contents and eventual cell death; for this reason, they are generally referred to as host defense peptides. Unlike conventional antibiotics that target metabolic processes, AMPs’ physical mechanism of action on lipid bilayers reduces the likelihood of bacteria developing resistance [10]. Their membrane-disruptive actions follow different models such as the carpet, barrel–stave, and toroidal-pore models, all of which rest on the initial AMP binding to the bacterial surface, followed by the formation of structural patterns that damage the membrane (Figure 1). In the barrel–stave model, peptides insert perpendicularly into the phospholipid bilayer, aggregating to form transmembrane pores that compromise membrane integrity [11]. The carpet model describes a mechanism in which peptides accumulate on the membrane surface, leading to its progressive disintegration [12]. Conversely, in the toroidal-pore model, peptides induce curvature in the lipid bilayer, forming pores composed of both phospholipid polar heads and the hydrophilic regions of the peptide [13]. Once integrated into the membrane, AMPs disrupt ionic homeostasis within bacterial cells, ultimately causing osmotic lysis and cell death. In addition to their membranolytic action, certain AMPs also target intracellular processes, interfering with cell wall biosynthesis, nucleic acid replication, and protein synthesis [14]. These complementary actions to membrane disruption make AMPs particularly effective against multidrug-resistant bacteria [10]. This multifaceted mechanism of action highlights the potential of AMPs as a promising class of antimicrobial agents in biomedical research. This review focuses on AMPs, exploring the diverse types and design strategies such as structural modifications and nanotechnology-based approaches to enhance their therapeutic efficacy. In brief, it also discusses preclinical and clinical bottlenecks that hamper translation from bench side to bedside use of AMP therapies.

## 2. AMPs’ Drawbacks and Challenges

Despite the numerous benefits of AMPs, some limitations still need to be addressed. Hemolysis represents the main drawback of AMPs due to their poor cell selectivity and high affinity toward human erythrocytes. The ability of AMPs to induce hemolysis depends on many different factors, including the balance between hydrophobicity and hydrophilicity; when this balance is in favor of hydrophobicity, it may facilitate the host cell membrane interaction and insertions, causing the pore formation and rupture. For instance, the AMPs Alamethicin and Melittin are endowed with a significant hemolytic activity because of their strong affinity against mammalian cells, determining the formation of transient or stable pores of the lipid bilayer [15,16]. Thus, clearly, the ratio between hydrophobicity and hydrophilicity is a key factor to keep in mind when designing new AMPs.

Another important drawback is the susceptibility of AMPs to proteases, making them unstable and low-potential agents for clinical studies. The L-amino acids are substrates highly recognized by proteolytic enzymes such as trypsin, chymotrypsin, and elastase. For instance, some linear natural AMPs, such as Magainin-2, Indolicidin, and Aurein 2.5, showed a low proteolytic stability against a wide range of enzymes [17]; in fact, these AMPs have undergone different chemical modifications for enhancing their antimicrobial potential. Moreover, this review describes several chemical strategies addressed to challenge AMPs’ disadvantages and improve their biological profile.

## 3. Structural Engineering Strategies for AMPs

Owing to the somewhat complex properties of AMPs and their key role in solving biomedical problems, scientists are continuously researching optimization strategies to overcome their shortcomings and improve their clinical application. This has led to several strategies, including but not limited to bioinformatic analysis, chemical modification, de novo design, and library screening [18].

### 3.1. Bioinformatic Methods for AMP Design

The integration of bioinformatics, computational tools, and data analysis enables the systematic design of AMPs with predefined properties by investigating amino acid position preferences, composition, and frequency. This approach leads to sequences with diverse amino acid compositions, structural conformations, and modes of action. Artificial intelligence (AI) and machine learning (ML) have emerged as efficient approaches to optimize AMP sequences by learning from extensive training datasets [19]. Additionally, bibliometric analysis, which uses statistical and mathematical techniques to quantitatively evaluate literature, plays a crucial role in tracking research progress, identifying emerging trends, and uncovering knowledge gaps [20]. Despite the growing interest in computer-aided AMP design, a comprehensive bibliometric analysis focused on this field within the context of infectious diseases is still lacking. Recently, a bibliometric analysis of computer-aided AMP design research from 2006 to 2024 has highlighted current hotspots and emerging trends, providing valuable guidance for future investigations in AMP therapeutics [21].

The comprehensive understanding of the physicochemical properties and structure-activity relationships (SARs) of AMPs has allowed researchers to predict and design novel peptides with predefined characteristics. Clearly, this process implies the use of computational tools and algorithms to evaluate the SARs of candidate peptides based on AMP-specific features, followed by the assessment of their potential antimicrobial activities [22]. Large datasets of known AMPs constitute the basis for AI and ML, which can predict numerous characteristics of new peptide sequences, facilitating the rapid design of potent AMPs. As a matter of fact, prediction of chemical properties allows not only to envisage biological activity and toxicity based on molecular structure but also to identify promising lead compounds for drug development [23]. For example, Lin et al. synthesized eight peptides by using the GAN-based AI model, and [24] showed a strong antibacterial activity against *Methicillin-Resistant Staphylococcus Aureus* (MRSA) and carbapenem-resistant *P. aeruginosa*, with MICs as low as 3 μg/mL. Similarly, the GAN model was also used by Tucs et al., where they generated and synthesized six peptides, and one of them exhibited very high potency (MIC ≈ 3.1 μg/mL against *E. coli*, roughly twice as strong as ampicillin) [25]. These examples illustrate how AI-based predictions can be successfully validated through reproducible in vitro experiments. Nonetheless, challenges regarding standardization and inter-laboratory reproducibility must be addressed to support broader clinical translation.

Furthermore, the development of several ML models further expanded the design and optimization of novel peptide sequences for the potential discovery of new therapeutic agents. In fact, ML models can foresee structural scaffolds or amino acid compositions with high affinity and activity against specific pathogens while minimizing harm to host cells or normal flora. This targeted design strategy accelerates the drug discovery process by identifying promising peptide candidates for further optimization and testing.

Deep neural networks (DNNs) are also a type of deep learning, increasingly used for predicting the antimicrobial and hemolytic activities and the therapeutic potential of AMPs [22]. DNNs can be trained on large datasets of known AMP sequences to produce new peptides and predict their activity against precise pathogens. A key advantage over traditional artificial neural networks (ANNs) is their multiple layers, which allow them to learn complex patterns within the data, resulting in more accurate predictions of antimicrobial and therapeutic properties.

Recent advancements in technology have accelerated the design and discovery of new lead compounds. AI is increasingly used to design high-efficacy AMPs by screening millions of available peptide sequences, identifying functional motifs and properties, and developing ML models that predict antimicrobial activity based on the peptide’s physicochemical properties.

### 3.2. Improving AMPs’ Physicochemical Properties

**Figure 2:**
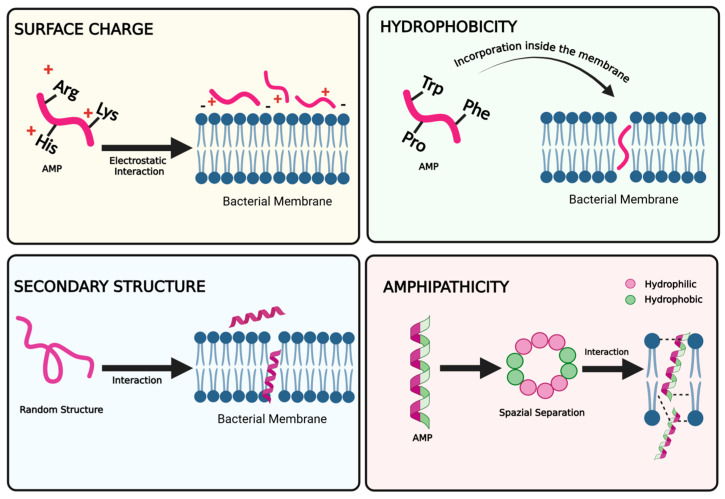
The classification of key physicochemical properties of AMPs influencing their membrane interaction and mechanism of action. The figure was created with https://app.biorender.com/ (Accessed on 20 June 2025).

The design of AMPs for therapeutic and biotechnological applications is mainly based on the understanding of their physicochemical properties; as a matter of fact, researchers continue to explore and optimize these properties. Rational design of AMPs involves tuning intrinsic properties that are key parameters in optimizing their antimicrobial efficacy. The cationic nature of many AMPs is a key property correlated to the presence of positively charged amino acids, which facilitates the initial electrostatic attraction toward negatively charged microbial cell surfaces (Figure 2); this interaction leads to bacterial membrane disruption and subsequent cell death. Thus, the inclusion of positively charged residues, such as lysines and arginines, can improve the cationicity of AMPs, enhancing their selectivity for bacterial membranes.

The amphipathic nature of many AMPs, characterized by spatially separated hydrophobic and hydrophilic faces within their molecular structure, is a crucial parameter. Thanks to their amphipathicity, they possess enhanced ability to insert into microbial membranes while retaining solubility in aqueous environments. As a matter of fact, some AMPs contain both cationic and hydrophobic amino acids, which presents scientists with a plethora of tuning opportunities. Falanga et al. [26] reported that hydrophobicity is a key concept for antimicrobial activity. In particular, the ability of AMPs to disrupt the cellular membrane, thereby altering several fundamental processes in microorganisms, is closely linked not only to their secondary structure but also to their hydrophobicity. In fact, it is well established that hydrophobic residues play a critical role in membrane interactions, contributing to both conformational stabilization and destabilization of lipid bilayers via the mechanisms mentioned above [27]. It is important to note that the subtle balance of cationic and hydrophobic residues is key to enhancing activity and selectivity toward bacterial membranes, and to reducing toxicity toward eukaryotic cells. Another key feature is their sequence length, typically ranging from 10 to 50 amino acids. The shortest sequences offer advantages in terms of cost-effective chemical synthesis, enhanced stability, and reduced immunogenicity. In addition, their diverse amino acid composition allows great flexibility in the design of peptides with specific properties, such as enhanced stability or selectivity against certain types of pathogens. Furthermore, many AMPs are also resistant to proteolytic degradation, enabling the preservation of activity in the presence of enzymes that would typically degrade peptides. Therefore, they are characterized by a significant longevity in the host and sometimes are also able to live in hostile environments. Aromatic residues, such as tryptophan, tyrosine, and phenylalanine, also play a vital role in facilitating peptide–membrane interactions and incorporation into the bilayer. Not only do they enhance the overall hydrophobic character of the peptide, thereby promoting its entry into cells, but also engage in specific π interactions with membrane lipids [28], and in fact, they are usually found at the interface between the lipid bilayer and the aqueous environment. Moreover, these residues can be easily incorporated into peptide sequences, which further improves their activity. The hydrophilic nature of peptides is crucial for ensuring their solubility in aqueous environments and for promoting interactions with the negatively charged components on the surface of microorganisms. Thus, fine-tuning the hydrophilic–hydrophobic balance of peptides is essential for optimizing their bioactivity [7].

Another factor driving the membrane interaction is the secondary structure of AMPs, influencing their effect on the membrane disturbance and selectivity [29]. The most common secondary structure adopted by AMPs is the α-helix, which facilitates their insertion into lipid bilayers. Proper helical folding is essential, as disruption of the helix can impair function. As said, this α-helical structure is typically facially amphiphilic, and such an organization is critical for membrane perturbation, particularly in AMPs that act through toroidal-pore or barrel–stave mechanisms. A key feature is their conformational plasticity, characterized by a change in the secondary structure when moving from the aqueous environment to the membrane bilayer. For example, the natural Temporin L (isolated from Rana Temporaria) and its analogues [30,31,32] adopt a random structure in aqueous environment, while they are completely structured in helical conformation in membrane-mimetic environments such as sodium dodecyl sulfate (SDS) micelles and liposomes mimicking bacterial membranes. This conformational transition is strongly correlated with their membrane-disruptive potencies and antimicrobial efficacy [33,34]. A similar behavior is observed also with the cathelicidin LL-37, which remains unstructured in aqueous solution but adopts a helical conformation upon membrane interaction that is correlated with its strong antimicrobial activity [35,36]. In addition to the helical structure, some AMPs adopt the β-sheet structure. Arenicin-3, isolated from the lugworm Arenicola marina [37], is an amphipathic β-hairpin antimicrobial peptide constrained by two disulfide bonds with a free N-terminal amine and C-terminal acid. This structural feature facilitates its interaction with and insertion into microbial membranes, leading to permeabilization and disruption through pore formation. Another interesting example is represented by human beta-defensins (hBDs), which present a mainly β-sheet amphipathic structure [38].

### 3.3. Chemical Approaches for Engineering AMPs

**Figure 3:**
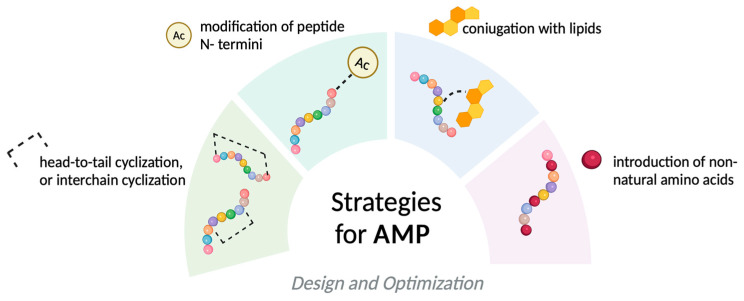
Representation of chemical strategies modification for AMP. The figure was created with https://app.biorender.com/ (Accessed on 20 June 2025).

To optimize AMPs for therapeutic use, various sequence modifications have been proposed to enhance antimicrobial activity and reduce toxicity [18]. Since only a small fraction of key amino acids are critical for antimicrobial activity, other residues can be modified or removed without compromising function. These modifications include backbone, side chain alterations, terminal modifications, amphipathic adjustments, cyclization, and lipidation (Figure 3).

For instance, a common strategy to increase AMP stability involves substituting L-amino acids with their D-counterparts, reversing stereochemistry to resist proteolytic degradation. D-amino acids are rare in nature and unrecognizable to proteolytic enzymes, thereby enhancing stability and antimicrobial activity. Similarly, incorporating non-natural amino acids, such as β-amino acids, modifies the backbone while preserving side chain identity, conferring protease resistance without diminishing function. A similar effect can be achieved through modifications of the peptide backbone. For instance, retro-inverso peptides are generated by reversing the sequence of amino acids from the N- to the C-terminus, resulting in structures that are both structurally and enzymatically stable.

Capping strategies, involving the modification of terminal or side chain residues, are effective approaches to improve the stability and antimicrobial activity of AMPs [18]. Common capping strategies include C-terminal amidation and N-terminal modifications such as acetylation, methylation, and lipidation, which reinforce the conformational stability of peptides, protecting them against enzymatic degradation, and often enhancing antimicrobial potency [18]. Furthermore, C-terminal amidation protects peptides from degradation by carboxypeptidases, while N-terminal acetylation or N-methylation prevents recognition by aminopeptidases, collectively increasing resistance to enzymatic cleavage and extending peptide half-life [39]. These post-translational modifications are integral to peptide-drug engineering, as they effectively improve the pharmacokinetic profile of AMPs.

Naturally occurring AMPs, such as cathelicidins, defensins, and aureins, are typically amidated at the C-terminus (–NH_2_), a modification closely associated with structural integrity and antimicrobial activity [40]. During amidation, the carboxyl group (–COOH) at the C-terminal is converted to a carboxamide group (–CONH_2_), either chemically or enzymatically. This modification not only increases stability but also reduces toxicity. For example, the C-terminal amidated Aurein 2.5 (GLFDIVKKVVGAFGS–CONH_2_) demonstrates significantly higher antimicrobial activity against Klebsiella pneumoniae compared to its non-amidated analogue [41]. Similarly, C-terminal amidation has been shown to stabilize helical structures, as seen in modelin-5-CONH_2_, which exhibits greater helical stability and enhanced efficacy against Escherichia coli than its non-amidated counterpart modelin-5-COOH [42]. Thus, terminal modifications are not merely structural refinements but are strategic interventions to enhance the therapeutic potential of AMPs, optimizing both stability and antimicrobial activity.

Halogenation is another potent strategy for optimizing AMPs. Incorporating halogen atoms such as chlorine, fluorine, bromine, or iodine can significantly enhance stability, solubility, and antimicrobial activity [43,44]. Fluorination, in particular, is widely employed in antimicrobial agents to improve pharmacokinetics. Drugs like Ciprobay (ciprofloxacin), Lipitor (atorvastatin), and Prozac (fluoxetine) utilize fluorine to modulate electrostatic properties, thereby improving efficacy [45]. The halogenation of jelleine-I, an AMP from honeybee venom, was proved to enhance both antibacterial and antibiofilm activities [46].

Cyclization also allows for the design of AMPs with tailored structural and functional properties. By selecting specific amino acid sequences and cyclization positions, it is possible to modulate the physicochemical properties of cyclic AMPs, optimizing their conformational stability, antimicrobial efficacy, and pharmacokinetic profiles. Cyclization involves converting linear peptides into cyclic peptides to achieve enhanced stability and resistance to proteolytic degradation [47]. This structural modification provides significant advantages, including increased stability both in vitro and in vivo. Additionally, cyclization reduces susceptibility to conformational changes that might otherwise diminish antimicrobial potency. Cyclic AMPs also demonstrate enhanced antimicrobial activity due to improved membrane penetration and peptide–membrane interactions [38]. One cyclization strategy involves head-to-tail cyclization using a beta-lactam moiety as a connecting bridge between the N- and C-termini. Head-to-tail cyclization imparts structural rigidity, reducing protease susceptibility. Additional stabilization can be achieved through disulfide or thioether bridges, which constrain peptide flexibility and improve circulatory stability. Other cyclization strategies involve forming amide or isopeptide bonds to produce closed-loop structures that are more resistant to enzymatic degradation and exhibit improved pharmacokinetics. For instance, a cyclic 17-amino acid β-defensin analog featuring a single disulfide bond was developed and characterized, and retained the antibacterial activity of the parent hBD1 and hBD3 against *P. aeruginosa*, *E. faecalis*, and *E. coli*, and exerts dose-dependent activity against herpes simplex virus type 1 [38].

Furthermore, lipidation strategies such as the attachment of fatty acids in the N-terminus or C-terminus of AMPs represent a significant promise [48]. In lipidation strategies, careful selection of the lipid tail length is critical, as the cytotoxicity of AMPs is influenced by the acyl chain length. Long lipid tails (i.e., C16 and C19) can compromise cell selectivity by increasing toxicity and may also reduce antibacterial activity due to a strong tendency to self-assemble in aqueous environments. Conversely, shorter lipid tails (such as C5, C8, and C10) enhance peptide hydrophobicity and facilitate insertion into lipid bilayers, while maintaining a balanced hydrophilic/hydrophobic profile that helps minimize toxicity. For example, Bellavita et al. demonstrated that conjugating C5 or C10 lipid tails to the N-terminus of Temporin L analogues enhanced their antifungal activity against Candida species, also producing synergistic or additive effects with voriconazole in resistant strains [32]. In contrast, Húmpola et al. reported that AMPs modified with longer lipid chains, such as C17 and C20, lacked activity against Candida species, likely due to their strong tendency to self-assemble in aqueous solution, which may hinder their interaction with fungal membranes [49].

Collectively, these structural modifications, summarized in Table 1, are crucial for addressing the inherent limitations of AMPs, particularly their high cytotoxicity and vulnerability to proteolytic degradation [38].

## 4. Biophysical Techniques for Studying AMPs Mode of Action

The binding, orientation, and positioning of peptides relative to lipid bilayers, along with lipid rearrangements induced by AMPs, are critical aspects of peptide-lipid interactions that play a key role in antimicrobial activity [50]. Various experimental techniques have been developed to study these interactions, differing in sample size, sensitivity, and the type and resolution of data they provide, offering complementary information.

Biophysical techniques like electron microscopy (EM), atomic force microscopy (AFM), and fluorescence imaging can reveal peptide-induced morphological changes. [51]. AFM uses lipid bilayer support to examine the AMP-induced changes in terms of membrane structure and thickness due to exposure to AMPs. For example, Lam et al. performed AFM by incubating the cationic AMP protegrin-1 on the zwitterionic support made of 1,2-dimyristoyl-sn-glycero-3-phosphocholine (DMPC) and observed the pore formation and wormlike micelles in the membrane [52].

Meanwhile, fluorescence imaging is highly sensitive but may involve dyes that could perturb the system and has limited resolution. Other methods, such as calorimetry, solid-state nuclear magnetic resonance (SSNMR), and X-ray analysis, can detect changes in the membrane’s thermodynamic properties and global phase transitions. SSNMR is a powerful technique, and two different approaches are used in characterizing the mechanism of AMPs. The first one consists of analyzing the structure, dynamics, and aggregation of 15N or 15N,13C isotope-labeled AMPs, in the presence of lipid environments mimicking bacterial membranes [53]. The second one is more sophisticated and examines the effects of AMPs directly on labeled living cells, and in this case, the bacterial cells are grown in the 15N,13C labeled medium [54].

Methods like fluorescence-based assays, electron paramagnetic resonance spectroscopy (EPR), infrared spectroscopy (IR), circular dichroism (CD), surface plasmon resonance (SPR), and X-rays can characterize both peptide and lipid structure and dynamics. IR, CD, and EPR are effective for determining peptide secondary structure, penetration depth, and orientation. Liquid-state nuclear magnetic resonance (NMR) uniquely provides comprehensive data on peptide topology and three-dimensional structure [55].

Fluorescence spectroscopy can aid in determining peptide orientation, position, and membrane affinity using fluorescent tags on AMPs or lipids. Liposomes, including Large Unilamellar Vesicles (LUVs) and Small Unilamellar Vesicles (SUVs) with defined lipid compositions, are commonly employed as biomimetic models of bacterial membranes [56].

**Figure 4:**
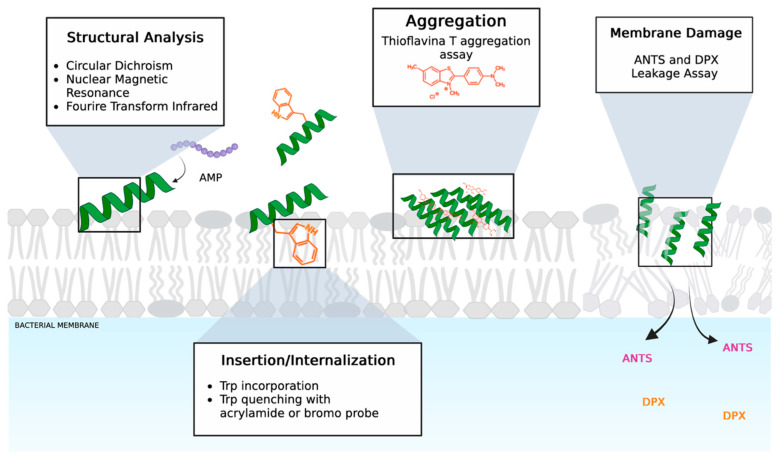
Cartoon representation of the structural change and hypothetical mechanisms of action of AMPs explored by some biophysical techniques including fluorescence-based assays and spectroscopic studies. The figure was created with https://app.biorender.com/ (Accessed on 20 June 2025). Computational approaches have also become essential for investigating peptide–membrane interactions, providing insights into dynamic processes at scales that experimental methods cannot easily access. Simulations have successfully predicted the insertion of helical peptides, although they face limitations due to challenges in accounting for peptide–peptide interactions and the complex environment within the bilayer.

Many biophysical studies exploit the intrinsic fluorescence of tryptophan (Trp), as AMPs often contain aromatic amino acids. Trp fluorescence is highly sensitive to hydrophobic environments, showing a blue shift and reduced intensity, indicating the insertion depth. To further probe membrane insertion, aqueous quenchers such as iodide or acrylamide [57], as well as brominated phospholipids [58], are employed to discriminate between surface-bound and deeply embedded Trp residues (Figure 4). Various fluorescence probes, including Thioflavin T, Laurdan, 8-aminonaphthalene-1,2,3-trisulfonic acid (ANTS), and p-xylylene-bis (pyridinium bromide) (DPX), can be used in different biophysical experiments to elucidate the mechanism of action of AMPs. For instance, ThT is widely used to monitor AMP oligomerization within bacterial membranes (Figure 4) [59,60], whereas Laurdan provides insights into membrane fluidity changes upon peptide interaction [61]. Liposomes encapsulating both ANTs and DPX are exploited to evaluate the pore-forming ability of AMPs [62]; upon membrane permeabilization, ANTS is released and dequenched from DPX, resulting in increased fluorescence intensity.

In addition, advanced techniques such as surface plasmon resonance (SPR) and Isothermal titration calorimetry (ITC) are employed to examine peptide–membrane interactions and binding. The SPR technique utilizes HPA (monolayer) and L1 (bilayer) sensor chips, providing quantitative data on binding kinetics and affinity constants that distinguish between surface adsorption and membrane insertion [63]. The Ka_bilayer_/Ka_monolayer_ ratio helps assess insertion depth, with ratios above ~10 indicating deeper membrane penetration. The ITC technique provides thermodynamic data on peptide binding, revealing secondary effects like membrane permeabilization and lipid phase transitions [64]. Differential scanning calorimetry (DSC) tracks lipid phase transitions, highlighting peptide-induced disruptions in lipid packing.

Furthermore, circular dichroism (CD) and NMR spectroscopies provide valuable insights into the secondary structure and orientation in membranes, enabling the effective analysis of peptide alignment and structural changes in lipid environments. CD spectroscopy, in particular, is widely used to monitor structural transitions of AMPs upon interaction with bacterial membrane models, typically in the presence of liposomes and across varying peptide-to-lipid ratios. Obviously, all these biophysical techniques, when combined, contribute to a detailed understanding of how AMPs interact with membranes and potentially disrupt or alter their structural organization. However, to accurately define the mechanism of action, it is essential to integrate these findings with complementary in vitro studies on bacterial cells. This integrated approach allows for a more comprehensive interpretation and supports the formulation of mechanistic hypotheses grounded in both molecular and cellular observations.

## 5. AMPs Conjugation Strategy

**Figure 5:**
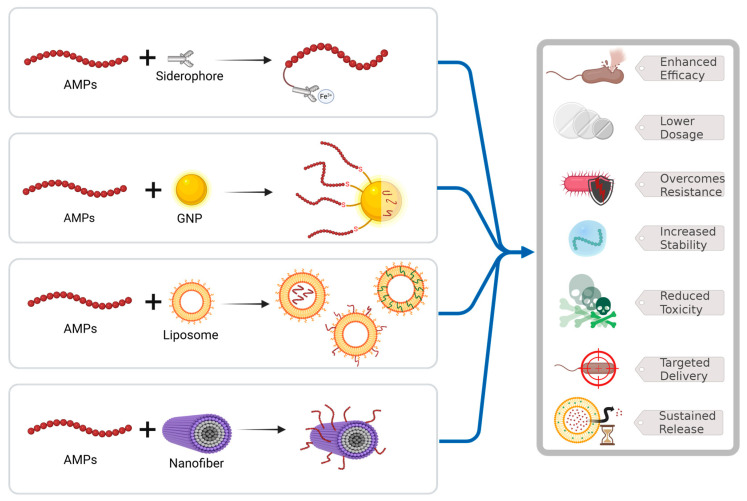
Strategies involving conjugation to boost the antimicrobial activity of AMPs. The figure was created with https://app.biorender.com/ (Accessed on 20 June 2025).

Conjugating AMPs to biological molecules or delivery systems aims to enhance their antibacterial efficacy and overcome the limitations associated with naturally occurring molecules, such as conventional antibiotics [65]. The covalent conjugation strategies typically involve chemical reactions such as click chemistry, amide bond formation between carboxylic acid and amine groups, or the reaction between cysteine residues and electrophilic groups like maleimides. These AMP conjugates offer several advantages, including improved stability, resistance to proteolytic degradation, enhanced safety, and prolonged half-life. In this review, we summarize three main conjugation strategies: the formation of metal–AMP complexes, the development of antibiotic–AMP conjugates, and AMP delivery via nanosystems (Figure 5).

### 5.1. Metal Chelator–AMP Conjugates

Metal-binding AMPs are considered an attractive class due to their unique ability to interact with metal ions, thus offering increased antimicrobial activity, which tends to ultimately lead to overcoming bacterial resistance. Several bacterial proteins require metal ions for optimal functionality; therefore, AMPs can interfere with and modulate metals’ roles, inducing bacterial death. Numerous metal ions, including Zn(II), Cu(II), Ni(II), and Fe(II), interact with AMPs that present binding motifs able to chelate specific metals. For example, some AMPs present the ATCUN motif (amino-terminal copper and nickel binding site) represented by the sequence H_2_N–X-X-His, able to bind Cu(II) and promote the production of reactive oxygen species (ROS) [66,67]. Additionally, the metal-AMPs binding cause variations in the peptide charge, structure, and mode of action, resulting in greater antibiotic efficacy [68]. Many studies suggested that the metal–AMP complexes tend to improve the affinity and permeabilization of AMPs for biological membranes, as well as their overall stability [48]. Another hypothesis underlying the mechanism of action of metal–AMP complexes is correlated with the metal sequestration by AMPs that deprive bacteria of these essential nutrients, ultimately leading to their death [68]. Clavanin A, which utilizes Zn(II) to stabilize its helical structure, is one of the numerous metal-binding AMPs that have been identified. This stabilized helical structure enhances its interaction with membranes [69]. Histatin 5 and Hepcidin-25 (involved in the regulation of iron homeostasis) use binding to Zn(II) and Cu(II), respectively, to increase their antifungal and antimicrobial activity. Another metal-conjugation approach consists of the incorporation of the metal chelator, such as siderophores, endowed with high affinity toward Fe(III), an essential nutrient to bacterial growth, as the bacteria acquire iron from the environment and from the host cells [70,71]. For instance, Bellavita et al. designed a synergistic multi-block molecule combining a modified antimicrobial peptide with an iron chelator via a bio-linker, demonstrating potential against Gram-negative pathogens through combined antimicrobial and iron-scavenging capabilities [72,73].

### 5.2. Antibiotic-AMPs Conjugates

Combining antibiotics and AMPs shows promise for boosting AMPs’ bioactivity and recovering antibiotic efficacy. This can be achieved either by covalently linking antibiotics to AMPs through various chemical conjugation methods or by co-loading both agents onto the same nanosystem [74], thereby amplifying their activity simultaneously [75].

The conjugation of AMPs with traditional antibiotics is an emerging approach aimed at overcoming antimicrobial resistance by leveraging the complementary mechanisms of action of both components. This synergistic strategy seeks to improve antimicrobial potency while potentially minimizing toxicity [76]. For instance, a vancomycin-cell-penetrating peptide conjugate, linked via 1,1′-carbonyldi-imidazole linker, exhibited strong antimicrobial activity against both resistant and non-resistant *S. aureus* strains, including methicillin- and Van-resistant staphylococcal strains. This conjugate disrupted bacterial cell integrity and demonstrated non-toxicity, suggesting its potential to overcome limitations in treating infections caused by these resistant strains [77]. Another example is the conjugation of UBI29–41 to chloramphenicol, which led to an enhancement of antibacterial effects against *S. aureus* and *E. coli* and a reduced toxicity [78]. Conjugation of AMPs to conventional antibiotics represents an evolving strategy to overcome the phenomenon of antibiotic resistance [79]. For example, the conjugation of β-lactam antibiotic precursors with AMPs has been investigated. Specifically, 7-aminocephalosporanic acid and 7-aminodesacetoxycephalosporanic acid were chemically modified and conjugated to the N-terminus of a series of cationic AMPs, improving their antibacterial activity, including against clinically isolated multidrug-resistant bacteria [80].

Another approach to fight resistance and restore the traditional antibiotic efficacy consists of synergistic AMP–antibiotic combinations. Exploiting the complementary mechanisms, AMPs disrupting membranes and antibiotics targeting intracellular pathways offer combinations that can enhance antimicrobial efficacy while lowering the required dosage of each agent. This synergy reduces the likelihood of toxicity and delays resistance development, offering a sustainable approach to infection management [81].

The effectiveness of AMP–antibiotic combinations depends on different complex parameters, including the kind of AMP and antibiotic, their concentrations and dosing regimens, and the type of resistance mechanisms. Recent research emphasizes that such combinatory approaches can revitalize the efficacy of older antibiotics, even against resilient multidrug-resistant (MDR) pathogens [37]. Mohammed et al. [82] demonstrated that FK16 exhibits bactericidal synergism with vancomycin, enhancing its antimicrobial activity against P. aeruginosa even at sub-inhibitory concentrations. Importantly, their study found that FK16 did not induce toxicity in human corneal epithelial cells or red blood cells, suggesting its potential as a safe adjunct to vancomycin therapy. In addition to demonstrating reduced toxicity and enhanced efficacy, existing studies suggest that combination therapy may improve biofilm penetration. Chernysh et al. [83] showed that Fly Larvae Immune Peptides (FLIP7), when combined with antibiotics, significantly lowered the required anti-biofilm concentration against *S. aureus* and *E. coli*. This effect was attributed to FLIP7’s ability to disrupt biofilms, thereby increasing antibiotic effectiveness. Furthermore, recent studies have demonstrated that these combination therapies not only enhance antibacterial efficacy but also suppress resistance development and even restore drug sensitivity in resistant strains. Particularly, Zhang et al. [84] explored this, evaluating the synergistic potential of two α-helical antimicrobial peptides (AMPs), K_4_ and K_5_, in combination with conventional antibiotics such as gentamicin, rifampicin, ciprofloxacin, and imipenem against Gram-negative bacteria. Their findings revealed that these combinations not only suppressed the development of antibiotic resistance but also restored sensitivity in previously resistant bacterial strains. The study demonstrated synergistic or additive antibacterial effects, particularly against drug-resistant pathogens.

### 5.3. AMPs’ Conjugation to Drug Delivery Tools

The conjugation of AMPs to a delivery system represents a promising strategy to enhance their stability and therapeutic efficacy. Optimizing AMPs for enhanced biological outcomes requires not only the development of potent biomolecules but also effective delivery mechanisms. The physiological environment of multicellular organisms often shortens AMP half-life due to enzymatic degradation and immune clearance, limiting their therapeutic potential. Advanced delivery systems are essential to overcome these challenges, ensuring stability, targeted delivery, and sustained activity at infection sites, thereby enhancing the clinical applicability of AMPs. To achieve specific and controlled release of AMPs, delivery systems can be functionalized with targeting ligands, such as antibodies or peptides, that bind to specific receptors or proteins on tissues or pathogens. AMPs can either be encapsulated within or conjugated onto the surface of these nanosystems, which offer a large surface area suitable for multi-functionalization [18]. These targeted systems not only increase the antimicrobial potency at the site of infection but also mitigate collateral damage to healthy cells [18].

The integration of AMPs into advanced delivery systems has become a cornerstone of modern therapeutic strategies, particularly against MDR pathogens [85]. Liposomal encapsulation and polymeric nanoparticles are central in this field, offering solutions to the inherent challenges of AMP therapies, such as instability, toxicity, and rapid degradation in physiological conditions. Liposomal systems, due to their biocompatibility and ability to encapsulate both hydrophilic and hydrophobic agents, have demonstrated remarkable potential in enhancing AMP bioavailability and ensuring sustained release at infection sites. For instance, studies have shown that liposome-encapsulated AMPs can reduce haemolytic activity while retaining antimicrobial efficacy, which is critical for minimizing off-target effects [86].

Nanoparticles composed of materials such as PLGA (poly (lactic-co-glycolic acid)) or chitosan have been employed to encapsulate AMPs, protecting them from proteolytic degradation while facilitating controlled release. Such systems are particularly advantageous for localized infections, as targeted delivery enhances the therapeutic index and reduces systemic toxicity [87].

AMPs can also be covalently conjugated to polymers: peptide–polymer conjugates (PPCs) can shield the positive charge of AMPs, improving their stability and solubility, which prolongs the in vivo circulation time and reduces cytotoxicity. PPCs have been applied in various medical fields, including wound dressing and antibacterial coatings. For example, polymeric nanoparticles like poly(lactide-co-glycolide) (PLGA) or gold nanoparticles (AuNPs) have been used to encapsulate AMPs, prolonging their antibacterial activity and reducing toxicity. For example, gold nanoparticles coated with indolicidin were developed and characterized in vitro for their dual function of preventing cell adhesion and eradicating biofilms formed by *Candida* clinical isolates and ATCC strains [88]. This preliminary study suggests the potential of the AuNPs–indolicidin complex as a therapeutic alternative for combating *Candida* biofilms, reducing ~70% of biofilm in comparison with the indolicidin alone, reaching 40% of inhibition at the same concentration [88]. Furthermore, to assess their nanotoxicity in mammalian cells and environmental organisms, the yeast *Saccharomyces cerevisiae* was used to evaluate growth, survival, genotoxicity, and expression of apoptosis. It was shown that *S. cerevisiae* is relatively resistant to AuNPs–indolicidin when compared to AuNPs and indolicidin alone. The lower toxicity exhibited by the AuNPs–indolicidin complex could represent an interesting feature from the perspective of its potential use for clinical applications [89].

Recent studies showed that self-assembling nanosystems present a promising strategy to engineer AMPs and increase their antimicrobial efficacy against diverse Gram-positive and Gram-negative bacteria, while reducing hemolysis, allergic responses, and resistance development [90]. The multivalent display of AMPs on the surface of nanostructures enhances peptide stability, extends half-life, and increases local peptide concentration, thereby improving antibacterial interactions and biocompatibility. Self-assembly is basically the process where molecules organize into ordered supramolecular structures through non-covalent interactions without needing any external push. This phenomenon is everywhere in nature and usually involves amphiphilic molecules. In water, this whole self-assembly business is controlled by a mix of forces: van der Waals interactions, hydrogen bonds, hydrophobic effects, electrostatic forces, and even π–π stacking come into play [91,92]. Amino acids, the building blocks of peptides, come with a whole variety of side chains (polar, nonpolar, charged, aromatic, etc.), which make them perfect for creating diverse non-covalent interactions [93]. This versatility is what makes them ideal candidates for engineering complex supramolecular architectures [94]. By fine-tuning parameters such as sequence length or side chain composition, it is possible to design a variety of nanostructures, ranging from vesicles and helices to nanotubes, sheets, nanorods, and even minimalist nanofibers [95]. Self-assembling peptides have shown promise in many areas: drug and gene delivery, tissue engineering, antibacterial therapies, vaccine development, catalysis, and even electronics. Among the most intriguing aspects of self-assembling peptides is their role as antimicrobial agents [96]. These peptides have been integrated into platforms for bacterial bio-imaging and biosensing [97], as well as into surface coatings that help the fight against infections [98]. Of particular interest, synthetic peptide derivatives further expand the range of possibilities, enabling the design of materials capable of disassembling, reorganizing, or even reassembling in vivo, thereby paving the way for entirely new antibacterial mechanisms [99].

There are several nanostructures based on self-assembling peptides that show intrinsic antimicrobial properties. For example, nanospheres form through symmetric peptide assembly and have hydrophobic cores that can encapsulate antimicrobial compounds for controlled release [100]. Peptide nanotubes, on the other hand, are cylindrical assemblies formed by the rolling of β-sheets. They are mechanically robust, and their surfaces can be functionalized depending on the therapeutic target; they can be engineered to be either rigid or flexible, with dimensions ranging from nanometers to micrometers [101,102]. Nanofibers are also widely studied. These elongated structures typically have a hydrophilic exterior and a hydrophobic core, optimizing their interactions in aqueous environments. A particularly striking example is provided by diphenylalanine-based nanofibers [103]. Even though these structures are minimalist, neutral, and entirely aromatic (which is quite different from the usual cationic or amphipathic antimicrobial peptides), they still exhibit impressive antibacterial activity and allow for extensive chemical modifications [104]. Indeed, research has demonstrated that diphenylalanine nanoassemblies can cause cell morphological disruption, stress-response pathway activation, bacterial growth inhibition, and even an increase in membrane permeability. By displaying AMPs on the surface of supramolecular nanostructures, researchers can boost the local concentration of active peptides, enhance stability, and prolong bioactivity, all while maintaining or even improving compatibility with human tissues [105].

The multivalent display of the antimicrobial peptide WMR within self-assembling nanostructures effectively enhanced antibiofilm activity against the antibiotic-resistant pathogens *P. aeruginosa* and *Candida albicans*. By exploiting WMR’s multivalent presentation, the nanosystem not only augments antibacterial efficacy but also serves as a platform for incorporating conventional antibiotics in a potentially pH-responsive, stimuli-sensitive delivery system. With favorable biocompatibility and biodegradability, these nanostructures present a promising strategy for the development of advanced antimicrobial materials [105].

Wang et al. [106] achieved a rational design and built up peptide-based fibrils with controllable degradation using enzymes. The AMPs used in this study, EAP2 and Melittin, were reported to be capable of self-assembly into a well-ordered, twisted fibrillar structure and could be degraded effectively in a controllable manner by gelatinase. These fibrils exhibited enhanced antimicrobial activity, with MIC values of 40 μM against *E. coli* and *P. aeruginosa*, compared to the EAP2 fibrils, which showed MIC values above 160 μM.

Cardoso et al. [3] reported the bi-functional rational design of Fmoc-peptides as both antimicrobial and hydrogelator substances. The tetrapeptide termed Priscilicidin was rationally designed for antimicrobial activity and molecular self-assembly into nanostructured hydrogels and exhibited potent antimicrobial activity against Gram-positive bacteria and fungi, including resistant strains, at low micromolar concentrations. The Priscilicidin self-assembled into extended nanostructures stabilized by Fmoc stacking and induced the bacterial death at lower concentrations of 17 μM on *S. aureus* and 8 μM on *C.* *albicans*. In the same vein, Miao et al. [107] designed a series of AMPs based on the structures of a synthetic AMP KFA3 and a naturally occurring host defense peptide substance P (SP) to obtain peptides retaining the high antibacterial activity of KFA3 and the immunomodulatory activity and low cytotoxicity of SP. In a different study by Wei et al. [108], the hybrid peptide CL was used to evaluate the therapeutic potential of enterohemorrhagic *E. coli* (EHEC) induced intestinal infection and intestinal inflammation. In vivo studies, mice in the C-L plus EHEC groups demonstrated less weight loss as compared to the EHEC group, and the microbiota composition improved significantly in mouse intestines. In addition, the hybrid peptide C-L relieves the clinical symptoms of mice by reducing tumor necrosis factor-α (TNF-α), Interleukin-6 (IL-6), and Interferon-γ (IFN-γ) and affects the MyD88 nuclear factor κB (NF-κB) signaling pathway to regulate the inflammatory response stimulated by EHEC.

All in all, the study and application of self-assembling peptides sit at a fascinating crossroads of chemistry, biology, and materials science. They offer versatile platforms for therapeutic innovation and hold promise in addressing some of the most challenging issues in modern medicine.

## 6. Preclinical and Clinical Studies on Engineered AMPs

While AMPs are primarily valued for their antimicrobial properties, their therapeutic potential extends further. For instance, AMPs derived from aquatic animals exhibit antiparasitic activity, targeting pathogens like *Leishmania braziliensis* and *Trypanosoma cruzi* [109]. Additionally, certain AMPs, termed anti-cancer peptides (ACPs), can selectively target tumor cells [9]. This specificity arises from the negatively charged phosphatidylserine on tumor cell membranes, induced by high levels of ROS and hypoxia, which alter the tumor microenvironment and disrupt membrane phospholipids [110]. AMPs also play an immunomodulatory role, bridging innate and adaptive immunity by influencing immune cells such as neutrophils, monocytes, and dendritic cells [111]. They stimulate cytokine and chemokine production, promoting inflammation and recruiting immune cells to infection sites. However, their ability to complex with self-DNA or RNA has been implicated in autoimmune responses, highlighting the duality of their function in immunity [2]. In essence, AMPs represent a versatile class of biomolecules, offering promising alternatives to conventional antibiotics and potential therapies for parasitic, cancerous, and immune-related conditions.

The clinical application of AMPs is limited by their hemolytic and cytotoxic effects at their antimicrobial concentrations or their lower biological activity in clinical environments. Another drawback includes their low proteolytic stability and consequent short half-life, and the ability of AMPs to bind serum proteins such as albumin, reducing their bioavailability.

**Figure 6:**
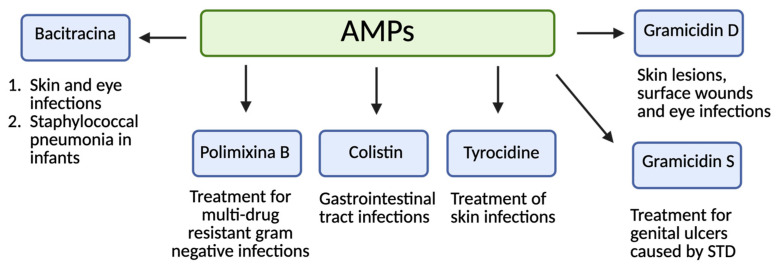
Approved AMPs for use in clinical settings.

A handful of AMPs have been approved for clinical use, which includes Colistin, Polymyxin B, Tyrocidine, Bacitracin, Nisin, Gramicidin, and Daptomycin among others (Figure 6) [10]. These linear and cyclic AMPs are widely used to treat resistant bacterial infections, either systemically or topically.

However, to date, most AMPs remain under scientific research development. There are still several peptide-based antimicrobial drugs that have entered the clinical trial stages, while some have been completed. For example, C16G2 was designed for dental applications; this AMP selectively targets Streptococcus mutans, which causes cavities. It has completed Phase 2 trials and could revolutionize dental care by preventing caries through selective microbial targeting [112]. Another AMP, hLF1–11, a fragment of human lactoferrin, exhibits activity against Gram-positive and Gram-negative bacteria as well as fungi. It has completed Phase 2 trials for patients with infections during neutropenia, demonstrating significant antimicrobial potential [113]. Brilacidin is a synthetic AMP mimetic that has demonstrated antibacterial, antifungal, and anti-inflammatory properties. It is under Phase 2 trials for conditions like oral mucositis and skin infections caused by resistant bacteria. Brilacidin disrupts bacterial membranes and may even act against biofilms [114]. In even more advanced phases of drug development, Phase 3, are Omiganan and Peceleganan Spray.

The antimicrobial peptide used in Omiganan is Omiganan pentahydrochloride, also known by its investigational code MBI-226. It is a synthetic cationic peptide derived from indolicidin, a natural AMP found in bovine neutrophils. Omiganan has been optimized to enhance its stability and activity against Gram-positive and Gram-negative bacteria, as well as fungi. Its mechanism involves disrupting microbial membranes, which results in the rapid killing of pathogens. Omiganan has been explored for use in preventing catheter-associated infections, treating rosacea, and addressing wound infections. Its strong antimicrobial activity and low propensity for resistance make it a promising candidate in clinical applications [115].

Peceleganan, also known as PL-5, is the antimicrobial peptide used in the Peceleganan Spray. It is a synthetic hybrid peptide derived from the combination of sequences from cecropin A (1–10) and melittin B (3–18). This hybrid structure enhances its antimicrobial efficacy and broad-spectrum activity against Gram-positive bacteria, Gram-negative bacteria, and drug-resistant pathogens [115]. Peceleganan is primarily intended for treating secondary wound infections, including diabetic foot ulcers (Wagner grade II), burns, abrasions, pressure ulcers, and other chronic wounds. Its novel mechanism of action makes it a non-antibiotic anti-infective drug, showing promising results in combating antibiotic resistance. Peceleganan has reached Phase II clinical trials in the U.S. and Phase III in China for diabetic foot ulcers and related conditions. In Table 2, a comparative analysis of selected clinical AMPs discussed in this section is reported.

However, the broader commercialization of AMPs remains challenging. Many AMPs in trials are natural or derivative forms that lack cutting-edge design improvements, and some of them fail because of their low therapeutic efficacy, poor proteolytic resistance, and side effects noted only in clinical trials. For instance, the AMP Pexiganan failed in Phase III for ulcer treatment because it did not show a superior effect in comparison with the traditional antibiotics, and its topical use requires high costs; hence, the cost–benefit ratio was not favorable [116]. Another example is represented by Iseganan (IB-367), an AMP derived from protegrin-1 that has failed during Phase III for the treatment of oral mucositis in cancer patients. Unfortunately, any improved effect was not observed compared to placebo, and side effects such as irritation in the mucosal area were noted [117].

While progress is being made, transitioning AMPs from research to clinical use will require ongoing innovation and optimization of their properties.

However, with over 3000 AMPs discovered [8], there are just about a handful of them clinically approved despite the obvious advantage they have compared to conventional antibiotics, one of which is a decrease in multidrug resistance. When designing drug delivery systems for AMPs, the primary goal is to selectively release peptide molecules so that they can be rapidly accumulated at the target site without affecting healthy cells. Simultaneously, to preserve peptide conformation, delivery systems must be crafted to elude the reticuloendothelial system, extend recycling, and diminish degradation rates [14]. The reasons for this under-utilization include both biological and technical upheavals. Challenges such as toxicity to host cells, tendency to cause haemolysis, susceptibility to proteolytic activity, as well as high production cost and stability issues continue to limit its usage. An ideal AMP should have high antimicrobial activity, low toxicity toward mammalian cells, high protease resistance and environmental stability, low serum binding capacity, easy accessibility, and low production costs [9].

## 7. Future Perspectives and Research Directions

There are still contending issues with the dominant use of AMPs in clinical applications. Some of these issues are scaling up production and reducing costs, overcoming resistance mechanisms to AMPs, and the integration with precision medicine and personalized therapies [118]. In terms of scale-up, the production of AMPs is often hampered by the high cost of chemical synthesis or recombinant production, requiring advanced biotechnological approaches to improve yield and reduce costs [119]. Innovations such as microbial fermentation and peptide mimetics are being explored to address these challenges. Furthermore, although AMPs are less prone to resistance than traditional antibiotics, there is evidence that certain pathogens can develop adaptive mechanisms, such as efflux pumps, biofilm formation, or changes in membrane composition, reducing AMP efficacy in prolonged use [120]. Lastly, the use of AMPs in precision medicine requires an understanding of patient-specific factors such as microbiome composition, immune status, and genetic variability, necessitating advanced diagnostics and tailored therapeutic regimens. The swiftness in addressing these issues will shorten the development lifecycle and put AMPs at the table of patients in its most desired form. Prompt and effective resolution of these challenges will significantly accelerate the development lifecycle of AMPs, ensuring their availability to patients in their most optimized and clinically effective form.

AMPs offer a promising alternative to traditional antibiotics, addressing the critical global challenge of AMR. With their broad-spectrum activity, unique mechanisms of action, and potential applications in various medical fields, AMPs stand out as versatile therapeutic agents. Advanced strategies, including rational design, efficient technologies such as artificial intelligence and machine learning, nanoparticle conjugation, and innovative delivery systems, are paving the way to overcome the challenges that limit their clinical application. The integration of AMPs into precision medicine and synergistic combinations with conventional therapies holds immense potential for personalized and effective treatments. While AMPs’ journey from research to clinical application remains difficult, ongoing advancements in design, optimization, and delivery mechanisms promise to unlock their full therapeutic potential. With continued innovation and interdisciplinary collaboration, AMPs could revolutionize infection management and contribute significantly to combating the AMR crisis.

## Figures and Tables

**Table 1:** A comparative summary of the AMPs’ modifications and their improvements.

Modification Type	Improved Properties	Antimicrobial Spectrum	Toxicity Profile
D-amino acid substitution	Protease resistance, structural stability	Broad spectrum	Reduced toxicity due to stability
β-amino acid incorporation	Protease resistance without loss activity	Broad spectrum	Low toxicity
Retro-inverso peptides	Protease resistance, increased half-life	Broad spectrum	Improved selectivity
Cyclization	Structural rigidity and stability	Broad spectrum	Lower toxicity
Lipidation	Improved membrane interaction and insertion	Broad spectrum including *Candida* spp.	Cytotoxicity due to reduced selectivity and strong aggregation propensity

**Table 2:** A comparative summary of the AMPs’ clinical development, efficacy, and limitations.

AMP	Source	Clinical Phase	Efficacy Outcome	Limitations
C16G2	Synthetic AMP	Phase II	Completed Phase 2; Effective in caries prevention	Limited to dental use; systemic effect unknown
hLF1-11	Human lactoferrin fragment	Phase II	Promising in neutropenic infections	Moderate bioavailability; risk of degradation
Brilacidin	Synthetic AMP	Phase II	Antimicrobial and anti-inflammatory effects	Under investigation
Omiganan	Synthetic AMP	Phase III	Strong efficacy; promising for catheter/wound infections	Limited systemic application
Peceleganan	Hybrid AMP	Phase III (China)	Effective in wound infections such as diabetic foot ulcers	Potential cytotoxic effect due to melittin-derived segment

## Data Availability

Not applicable.
